# Case report: Perioperative management of a patient with shapiro syndrome during on-pump cardiac surgery

**DOI:** 10.3389/fcvm.2023.1250110

**Published:** 2023-09-19

**Authors:** S. Boskovic, V. Ciobanu, N. Theuerkauf, F. Bakhtiary, M. Velten

**Affiliations:** ^1^ Department of Anesthesiology and Intensive Care Medicine, University Hospital Bonn, Bonn, Germany; ^2^ Department of Cardiac Surgery, University Hospital Bonn, Bonn, Germany

**Keywords:** temperature management, minimal invasive aortic valve replacement surgery, spontaneous periodic hypothermia, shapiro syndrome, temperature

## Introduction

In 1969 Shapiro et al. described a specific triad, consisting of episodic hypothermia, hyperhidrosis, and corpus callosum agenesis (1). Less than 80 cases have been reported till today and the pathophysiological mechanism of this rare disorder remains to be elucidated. Hypothermic episodes differ in duration, frequency, and most important severity between patients. Current expert opinions on the topic involve a dysregulation of the hypothalamic body “thermostat”. The initial drop from base line of 37°C (98.6°F) to a lower degree results in hyperhidrosis. Commonly, patients experience chills as the body temperature rises during recovery to regular body temperature (2). Other than the typical triad, a wide variety of different symptoms including headaches, hypoglycemia, or changes in blood counts have been reported (3–5).

Core body temperature is one of patient’s vital signs that is closely monitored during anesthesia. Hypothermia, being defined as a core body temperature below 36°C (96.8°F), occurs in 20%–70% of all patients during surgical procedures, potentially leading to arrhythmias, blood clotting disturbances, changes in pharmacokinetics of drugs, and many more complications (6).

Herein, we report a case of a patient with known Shapiro syndrome, undergoing endoscopic aortic valve replacement for aortic stenosis.

## Case report

A 59-year-old woman (59 kg, 163 cm) with severe aortic stenosis presented to the authors’ institution for elective minimally invasive, endoscopic aortic valve replacement surgery. Cardiac assessment revealed a sclerotic aortic valve with a high-grade stenosis, a valve area of 0.55 cm^2^, and peak velocity of 3.85 m/s, without regurgitation or dilatation of the ascendence aorta. Left ventricular function was preserved with an ejection fraction of 64% and a minimal mitral valve regurgitation was observed.

The Shapiro syndrome was diagnosed two years prior to the scheduled cardiac surgery as the patient experienced an episode of hypothermia with a reported body core temperature of 30.7°C (87.26°F) resulting in ventricular fibrillation and cardiac arrest that was treated by cardiopulmonary resuscitation (CPR). After recovery, the patient received an implantable cardioverter-defibrillator (ICD Medtronic Miro VR) and a drug therapy with 200 mg clonidine daily was initiated as a prophylactic measure to which the patient responded well with no further occurrences of hypothermia since then. Going through detailed patients’ medical history it was revealed that she had so called episodes of low body temperature all her life, sometimes as low as 29°C (84.2°F) with profuse sweating, vomiting, and diarrhea, followed by chills as the body temperature rose back to 37°C (98.6°F). She personally had contributed these episodes to psychological disturbances and did not seek medical advice. The diagnostic following CPR revealed an aortic valve stenosis of 1.1 cm^2^. Neither the electrocardiogram (ECG), nor cardiac magnetic resonance imaging (MRI) revealed further pathologies. There were signs of a mild coronary artery disease, hypoplasia of the left vertebral artery, essential hypertension, and fibromyalgia. The brain MRI revealed a pathological contact between the acoustic nerve and a small blood vessel on the left side with no clinical correlation and no further pathological changes. Prior to surgery her body mass index was 22.2 with a body surface area of 1.63 m^2^. There were no abnormalities in laboratory findings. The patient is a smoker and married.

She had progression of her aortic valve stenosis through regular close follow ups. Two years after the initial diagnosis the indication for an aortic valve replacement and decision for a minimally invasive surgery procedure were made.

As a precautionary measure the patient was placed on a full underbody air warming blanket (Mock) upon arrival in the operating room (OR) for potential perioperative temperature control if needed. Prior to induction of general anesthesia, the patients’ body temperature was 37°C (98.6°F) and an arterial line (Vygon) was introduced for invasive blood pressure monitoring. Following general anesthesia induction with 100 μg remifentanil, 70 mg propofol, and 50 mg rocuronium the patient was intubated using a 7.5 mm [ID] endotracheal tube. Ultrasound guided internal jugular vein cannulated was performed, a four-lumen central venous line (Arrow) and a 9Fr sheath introducer (Arrow) were introduced, a urinary catheter with a temperature sensor (Resch) and a second rectal temperature probe (Resch TeleFlex) were placed for a more comprehensive temperature monitoring. Anesthesia was maintained using sevoflurane and a continuous infusion of remifentanil. Further monitoring consisted of ECG, capnography, peripheral oxygen saturation, central venous pressure, near-infrared spectroscopy (Medtronic), bispectrality index (Medtronic), and transesophageal echocardiography (GE Healthcare). The operating theater thermostat was set at 22°C (71.6°F), preventing temperature loss.

**Figure 1:**
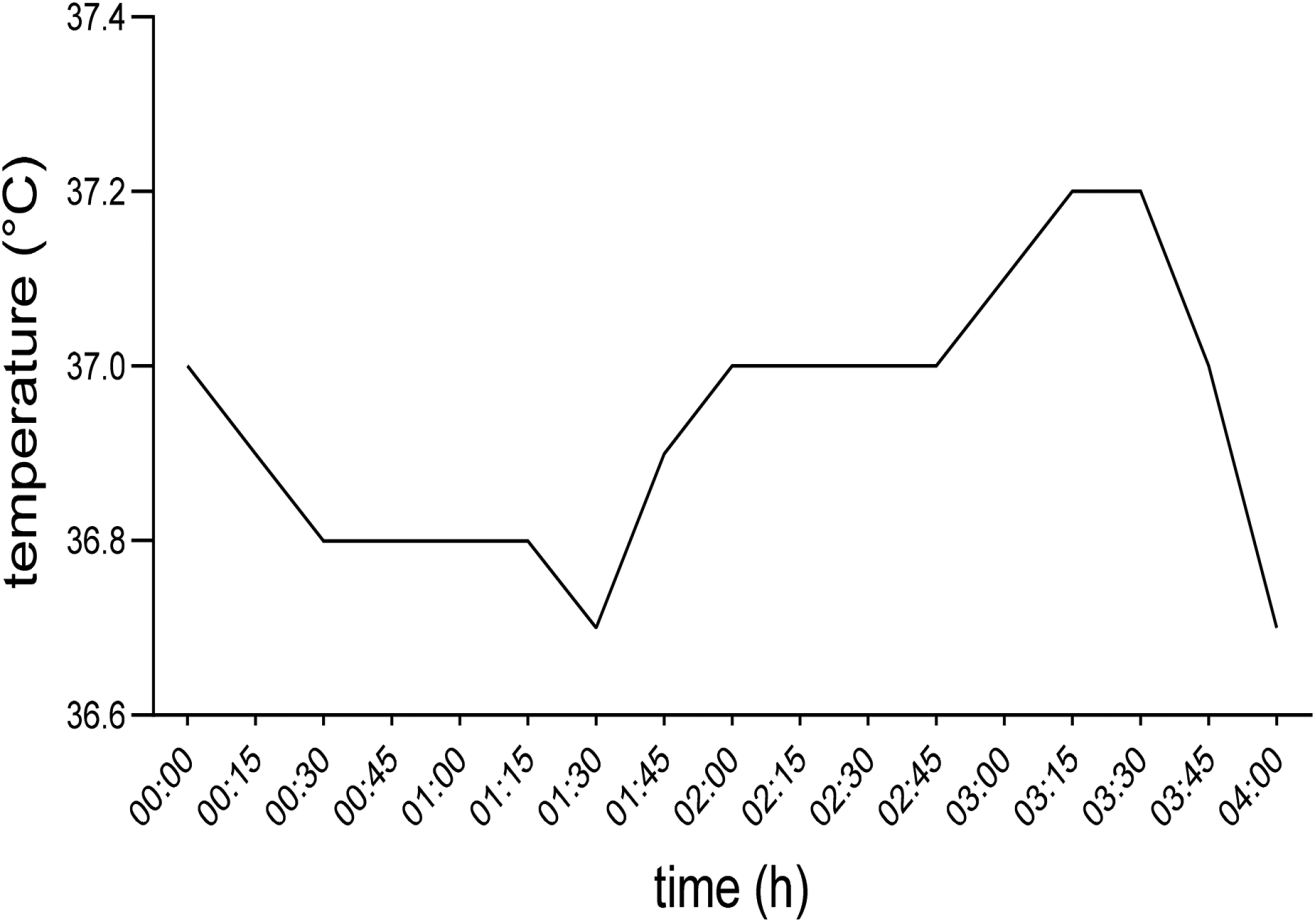
Body core temperature of the patient during surgery. 00:00 timestamp indicating the first measurement before inducing general anesthesia.

The procedure was started after pre-surgical TEE confirming of the diagnosis. Peripheral canulation was performed through the right femoral artery and vein. Bypass was initiated after TEE confirmation of venous canula positioning in the superior vena cava. Surgical approach to the aortic valve was through an anterolateral thoracotomy 6 cm in length. Before initiating CPB the patient's body temperature gradually reduced from 37°C (98.6°F) at the beginning of anesthesia induction to 36.8°C (98.24°F) over the course of 87 min and therefore no active warming was initiated. During CPB the patient was actively warmed through the by-pass machine (Stocking S5) to a body temperature of 37.0⁰C (98.96°F). During CPB the patient required high vasopressors dosages (up to 1.7 μg/kg/min noradrenalin and 4 U/h vasopressin) to establish a perfusion pressure above 65 mmHg. As prophylactic measure due to an anticipated strong systemic inflammatory response syndrome the patient received 100 mg of hydrocortisone and to shorten the ischemic and reperfusion time a biological, sutureless, self-expanding Percival Plus M size replacement aortic valve was implanted. The left atrial auricule was closed with a 35 mm AtriClip. Overall CBP time was 104 min with 59 min cross-clamp and 11 min reperfusion duration (Figure 1).

After the patient was weaned from cardiopulmonary bypass her hemodynamic state was gradually recovering, while she slightly cooled down and over the course of 45 min reached a body temperature of 36.7°C (98.06°F). Prior to the end of the surgery, the patient received 7 mg piritramide, 1,000 mg metamizole as postoperative analgesia, 50 µg clonidine and 4 mg ondansetron as postoperative nausea and vomiting prophylaxis, according to institutional standards. Following uncomplicated extubation, 17 min after suturing, the patient was transferred to the intensive care unit (ICU) on low dose noradrenalin (0.05 µg/kg/min) (Figure 1).

**Figure 2:**
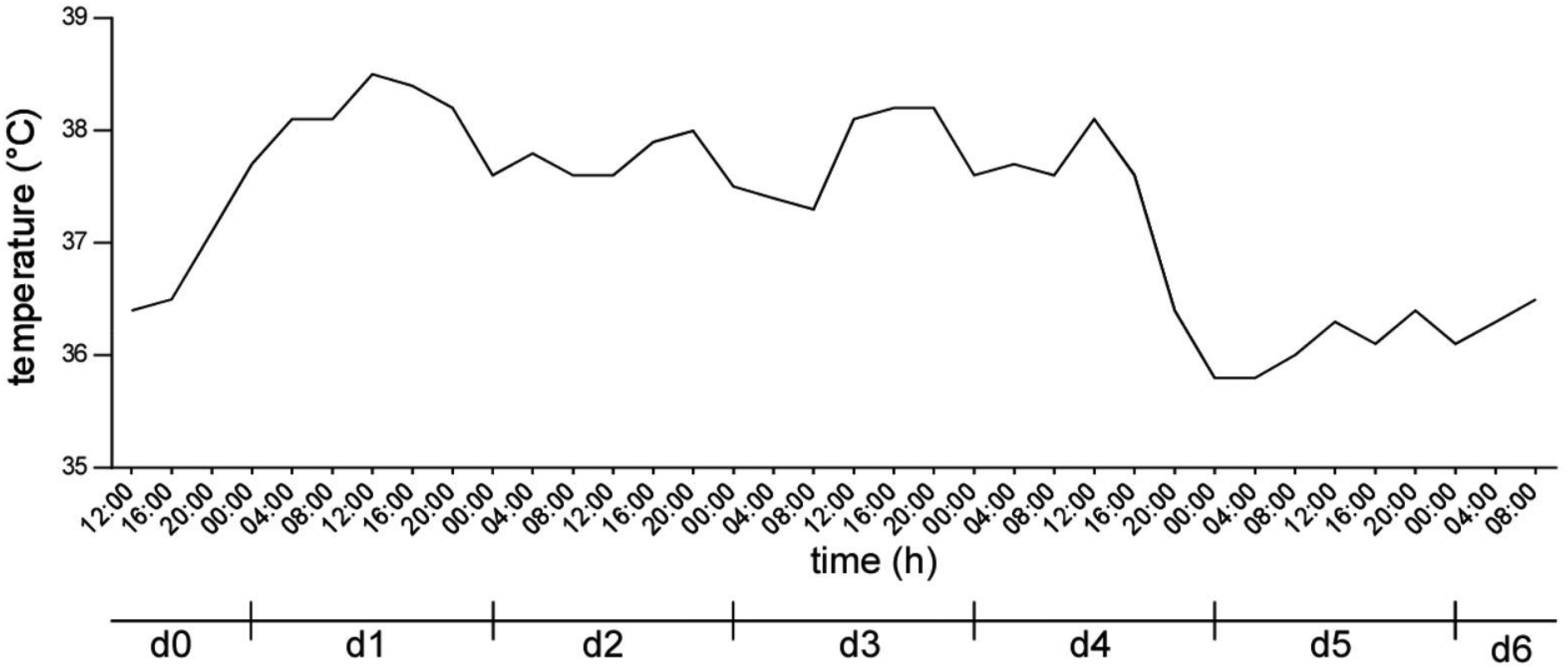
Body core temperature of the patient after surgery. d0 indicating the day of surgery, d1 to d6 indicating the first to sixth postoperative day.

On the first evening after the surgery the patient started experiencing fevers peaking at 38.5°C (101.3°F) and therefore remained on ICU observation. Elevated body temperature continued on the second postoperative day and the patient experienced an asthma attack from which she quickly recovered. Other than flatulence the patient had no other complaints during the postoperative course and as her vital signs stabilized with body temperature normalizing, she was transferred to the ward on the fourth postoperative day. On the sixth postoperative day the patient was discharged to further outpatient care with a body temperature of 36.5°C (96.8°F) and without any complications. During her entire hospital stay the patient had no episodes of hypothermia (Figure 2).

At her scheduled 1 month and 3 months post-op follow-up the patient was feeling well and had no occurrences of hypothermia. Echocardiography showed a preserved ejection fraction of 64.1%, no evidence of paravalvular leakage and a good quality function of the implanted aortic valve with Vmax. of 2.3 m/s.

## Discussion

Shapiro Syndrome is an extremely rare medical condition and through our extensive research of the available literature up until our case report there have only been 78 cases reported with just one during general anesthesia and none during cardiac surgery.

Our patient was diagnosed with Shapiro Syndrome after an incident of ventricular fibrillation and CPR. Atrial and ventricular dysrhythmias are a common life-threatening complication of hypothermia. In approximately 20,000 cases in the UK and 25,000 cases in the USA hypothermia was either the leading or an attributing cause of death annually (7, 8).

The typical triad of Shapiro Syndrome is episodic hypothermia, hyperhidrosis, and agenesis of corpus callosum. In the extensive diagnostics post cardiac arrest, our patient lacked the absence of corpus callosum agenesis, but 50% of all patients with Shapiro syndrome have no corpus callosum abnormalities, 40% have a complete agenesis, and 10% have other anatomical changes in the region. Her episodes of hypothermia were followed by gastrointestinal disturbances which occurs in around 14% of patients with Shapiro Syndrome (9).

Body temperature drops in Shapiro Syndrome patients are related to an disbalance between the anterior and posterior hypothalamus centers. The first being heat-dissipating and the second being heat conserving. There are various theories for this disbalance (degenerating, irritating, neurochemical, epileptical) but none are conclusive as to why the hypothalamus temperature set point is altered (10). General anesthesia damps thermoregulatory responses such as shivering or vasoconstriction caused by hypothermia and with non-shivering thermogenesis having little role in adults, patients become somewhat poikilothermic during surgery (11). Our case report showed that general anesthesia for cardiac surgery and CBP does not trigger hypothermic episodes in a Shapiro Syndrome patient.

An inflammatory response to CPB is well documented and increases in interleukin-6 have been reported (12). It is believed that this activation of the immune system is being generated as a result of its’ contact with the artificial surface of the CPB machine, as well as genetic factors, which also play a major role (13, 14). Clinically, our high vasopressor dosage requirement during CPB potentially resulted from a strong inflammatory response during CPB and our patient was given glucocorticoids, with a good outcome and a fast reversal of vasoplegia, although their role in preventing or damping the inflammatory response during CBP remains questionable (15).

The anterolateral thoracotomy approach was chosen for our patient as newer studies have shown that the inflammatory response to CBP is lower in using this approach rather than median sternotomy (16). This approach is also linked to a shorter ICU and hospital stay, reduced infection rate and less blood loss (17). The cross-clamp time was shortened using a sutureless self-expanding prosthesis. Although self-expanding prosthesis are linked to a higher incident of postoperative permanent pace maker implantation, our patient already had an implanted ICD (18). Minimal invasive endoscopic aortic valve replacement is very suitable for middle-aged patients with preserved ejection fraction, which is the case regarding our patient (19).

Postoperative hyperthermia after surgery occurs in more than 25% of patients after major surgery which peaks at approximately 12 h after surgery and is associated with elevated levels of interleukin-6, which through inflammatory responses sets a new value of the thalamic thermostat set-point (20, 21). Our case report showed that patients suffering from Shapiro Syndrome react to inflammatory interleukins similar to what is described in the literature (20, 21) and that although the thalamic thermostat episodically sets to a new low point it is possible to be set to a new high point through inflammation.

Clonidine medication was not paused preoperatively, with the patient taking her medication as per usual on the morning of surgery and continued immediately after surgery as well as the patient receiving a small dose of 50 µg clonidine prior to extubation. With such a rare disorder as Shapiro Syndrome there is an obvious lack of guidelines for treatment. Most commonly, patients are treated with cyproheptadine, clonidine, or carbamazepine. Patients treated with clonidine show either a full recovery with no new episodes of hypothermia or a drastic reduction of instances and duration of symptoms (2).

Up until our case report, to the best of our knowledge, there has been only one case of a patient with Shapiro Syndrome undergoing surgery and general anesthesia. The patients’ body temperature regulation reacted similar to other patients undergoing the same surgical procedure (22).

## Conclusion

This case report shows that Shapiro syndrome patients need to be closely monitored during the peri operative period, and investigate all the options to support the temperature homeostasis. Our case demonstrates this is doable and successful through close monitoring. More studies are needed though to understand temperature homeostasis.

## Data Availability

The original contributions presented in the study are included in the article/Supplementary Material, further inquiries can be directed to the corresponding author.
